# Time-Adaptive Machine Learning Models for Predicting the Severity of Heart Failure with Reduced Ejection Fraction

**DOI:** 10.3390/diagnostics15060715

**Published:** 2025-03-13

**Authors:** Trevor Winger, Cagri Ozdemir, Shanti L. Narasimhan, Jaideep Srivastava

**Affiliations:** 1 Department of Computer Science & Engineering, University of Minnesota, Minneapolis, MN 55455, USA; 2 Center for Learning Health System Sciences, University of Minnesota, Minneapolis, MN 55455, USA; 3 Department of Computer Science & Engineering, University of North Texas, Denton, TX 76205, USA; 4 Division of Pediatric Cardiology, Masonic Children’s Hospital, Minneapolis, MN 55454, USA

**Keywords:** heart failure with reduced ejection fraction, passive-aggressive classifier, machine learning, personalized machine learning, time-adaptive machine learning

## Abstract

**Background:** Heart failure with reduced ejection fraction is a complex condition that necessitates adaptive, patient-specific management strategies. This study aimed to evaluate the effectiveness of a time-adaptive machine learning model, the Passive-Aggressive classifier, in predicting heart failure with reduced ejection fraction severity and capturing individualized disease progression. **Methods:** A time-adaptive Passive-Aggressive classifier was employed, using clinical data and Brain Natriuretic Peptide levels as class designators for heart failure with reduced ejection severity. The model was personalized for individual patients by sequentially incorporating clinical visit data from 0–9 visits. The model’s adaptability and effectiveness in capturing individual health trajectories were assessed using accuracy and reliability metrics as more data were added. **Results:** With the progressive introduction of patient-specific data, the model demonstrated significant improvements in predictive capabilities. By incorporating data from nine visits, significant gains in accuracy and reliability were achieved, with the One-Versus-Rest AUC increasing from 0.4884 with no personalization (zero visits) to 0.8253 (nine visits). This demonstrates the model’s ability to handle diverse patient presentations and the dynamic nature of disease progression. **Conclusions:** The findings show the potential of time-adaptive machine learning models, particularly the Passive-Aggressive classifier, in managing heart failure with reduced ejection fraction and other chronic diseases. By enabling precise, patient-specific predictions, these approaches support early detection, tailored interventions, and improved long-term outcomes. This study highlights the feasibility of integrating adaptive models into clinical workflows to enhance the management of heart failure with reduced ejection fraction and similar chronic conditions.

## 1. Introduction

Heart failure with reduced ejection fraction (HFrEF) is a chronic, progressive condition affecting the heart muscles’ pumping ability. It is characterized by the heart’s inability to supply sufficient blood flow to meet the body’s needs, leading to symptoms such as fatigue, breathlessness, and fluid retention [1]. HFrEF poses a significant challenge within the global health landscape, affecting millions worldwide and markedly impacting morbidity, mortality, and healthcare systems [2]. The economic burden of HFrEF on healthcare systems is substantial, with the estimated average cost of hospitalization for HFrEF ranging from USD 3780 to USD 34,233 [3].

Machine learning has significantly advanced the prediction and diagnosis of HFrEF, increasing prognostic capabilities within the medical system across various levels of data fidelity. The heterogeneity of data sources in HFrEF prediction studies underscores the disease’s multifaceted nature and the potential of utilizing diverse data modalities for various prediction problems. Among data sources, ECG stands out, with high-performance models developed to identify patients with HFrEF [4,5,6], determine the severity of HFrEF [7], or distinguish normal heartbeats from those affected by HFrEF [8], realizing the promise of a high-fidelity cardiovascular measure in HFrEF prediction tasks. Another frequently used data source is Electronic Health Records (EHR), which provide a semi-comprehensive view of a patient’s health history by keeping a record of touch-points at various interactions with the healthcare system, e.g., doctor visits, laboratory measurements, etc. EHR data have been utilized to address a large number of prediction problems related to HFrEF, such as distinguishing individuals with HFrEF [9,10], assessing HFrEF severity [11,12,13,14], analyzing the survival of HFrEF patients over periods of time [15], and predicting hospital readmission among HFrEF populations [16,17]. Emerging data sources such as speech signals [18] and H&E-stained whole-slide images [19] illustrate the expanding landscape of HFrEF research, highlighting the role of integrating multi-modal data in enhancing predictive accuracy and adding to the diversity of clinical prognostic and diagnostic applications.

There are many traditional clinical methods to quantify the severity of HFrEF, each with limitations. The subjective New York Heart Association (NYHA) classification categorizes patients based on self-reported physical limitations [20]. The Weber Classification, using cardiopulmonary exercise testing (CPET) to measure peak VO2, offers objective functional capacity assessment but requires specialized resources [21]. Risk scores provide additional stratification: the Barcelona Bio-Heart Failure (Bio-HF) Risk Score predicts short-term mortality after acute events using clinical variables and biomarkers [22], while the MAGGIC HF Risk Score estimates long-term mortality in patients with chronic HFrEF using readily available clinical data, excluding biomarkers [23].

The complexity of HFrEF, characterized by its varied etiologies and patient presentations, necessitates a shift towards more personalized and predictive healthcare models [24,25]. The advent of large-scale health datasets, alongside breakthroughs in machine learning and analytical capabilities, provides unprecedented opportunities to tailor interventions and improve outcomes for HFrEF patients [26,27]. This study utilizes the National Institute of Health’s All of Us Research Program’s extensive and diverse datasets to develop time-adaptive and personalized machine learning models for predicting HFrEF severity. The All of Us initiative aims to gather health data from over a million people across the United States to enhance our understanding of health and disease [28]. By utilizing clinical visits of patients managing HFrEF, we aim to identify both individual and temporal patterns indicative of the severity of each patient’s HFrEF at each time point.We believe that such a model could not only further improve patient outcomes but be utilized by clinical care teams over time, offering more personalized care and assisting in facilitating earlier interventions. This research prioritizes the development of minimally intrusive (modest patient history, basic demographic data, no genomic sequencing, etc.) high-performing and personalized models. We believe models like the one proposed in this study can integrate smoothly into the current healthcare continuum, providing accurate predictions while minimizing patient risk and systematic burden.

## 2. Materials and Methods

### 2.1. Dataset Description

The All of Us Research Program represents a significant step forward in precision medicine. Launched to gather health data from one million or more people living in the United States, the initiative aims to advance individualized prevention, treatment, and care for people of all backgrounds [28]. Participants in the program contribute a broad range of data about their health, lifestyle, and environment over time. The All of Us dataset is distinctive for both its diversity and its scale. It encompasses many biological samples, EHR, and self-reported questionnaires [28]. One of the dataset’s key strengths is its commitment to representing groups that have historically been underrepresented in biomedical research.This inclusion ensures that the findings and advancements resulting from the All of Us program have the potential to benefit a more diverse set of the population, hopefully reducing health disparities and achieving a more equitable healthcare system. Offering researchers access to a comprehensive and diverse dataset, the All of Us Research Program has the potential to pave the way for a gold standard dataset for the future of precision and personalized medicine.

#### Data Selection, Processing, and Imputation

We identified individuals diagnosed with HFrEF (SNOMED Concept ID: 319835), resulting in an initial cohort of 11,800 participants. In order to construct a holistic profile of our selected cohort, we extracted demographic, physiological, laboratory-based, and medication data. The demographic data extracted includes date of birth and sex at birth, alongside self-identified parameters such as gender, race, and ethnicity. The physiological measurements we selected include only height and weight; from this, we could compute Body Mass Index (BMI)—a key indicator of general health and obesity levels, which are significant risk factors for HFrEF. Additionally, we included frequent clinical measurements of systolic and diastolic blood pressure readings; these signals provide additional insights into the cardiovascular strain experienced by participants. Laboratory-based measurements were also selected, including A1c levels, urea nitrogen, and Brain Natriuretic Peptide (BNP) levels. These biomarkers offer a window into participants’ metabolic and heart function status, aspects integral to managing and understanding HFrEF’s progression and impact. Medication data are also included in our dataset. We selected medication lists for each participant and investigated therapies closely related to the treatment and management of HFrEF, including beta blockers, angiotensin receptor blockers (ARBs), calcium channel blockers (CCBs), diuretics, ACE inhibitors, and statins.

The initial step in the selection criteria for our datasets was to ensure that BNP had been measured at least ten times for each participant. We wanted to ensure the dataset reflected individuals with substantial HFrEF monitoring. Accounting for the varied timing of measurements (clinical, in-laboratory, etc.), we introduced a 14-day window surrounding each BNP measurement for other data to be included in that measurement vector. This adjustment allowed the incorporation of related health indicators within close temporal proximity to each BNP measurement, aiming to provide a more accurate representation of each patient’s health status at the time of the BNP measurement. We utilized mean imputation at the patient level based on previous clinical measurements to address missing data. Compared to other imputation methods, this methodology for imputation kept patients’ values more personal and less reliant on other patients’ data and was only reliant on past data, simulating what would be available in a clinical setting. After imputation, patients who still had data missing from their records were excluded. After applying these filtering steps, the final dataset consisted of 1312 participants, each having at least 10 BNP readings. Table 1 shows the characteristics of the final population utilized in this study.

We utilized modest feature engineering methods to create our feature set for modeling, aiming to enrich our dataset further. We converted participants’ birth dates and BNP measurement dates to compute an ‘age at measurement’ feature, acknowledging age’s role in HFrEF management and the underlying dynamics. Additionally, we computed BMI from height and weight measurements to explore obesity’s impact on HFrEF, given its significance as a risk factor. We further transformed our dataset by identifying participants’ usage of various medications, marking the presence of these treatments as binary features according to treatment type, i.e., beta blockers, ARBs, CCBs, diuretics, ACE inhibitors, and statins. Table 2 shows the final feature set used for model training.

### 2.2. Clinical Measurements

#### 2.2.1. A1C

A1c (glycated hemoglobin) measures the average blood glucose control over two to three months and is a key indicator in diabetes management [29]. Research indicates a significant correlation between elevated A1c levels and an increased risk of (HFrEF), as poor glycemic control can lead to cardiovascular complications [30]. In particular, high A1c levels are associated with cardiovascular diseases because they promote arterial stiffness, microvascular disease, and atherosclerosis, thereby increasing the risk of HFrEF among individuals with diabetes or elevated levels of blood glucose.

#### 2.2.2. Urea Nitrogen

Urea nitrogen levels, commonly measured through the blood urea nitrogen (BUN) test, assess kidney function by gauging the amount of nitrogen in the blood that comes from the waste product urea. Urea is formed when proteins break down in the liver, and healthy kidneys filter urea out of the blood, excreting it in the urine. Elevated BUN levels can indicate impaired kidney function, dehydration, or a high-protein diet, while low levels may suggest malnutrition or severe liver damage [31,32]. HFrEF can lead to decreased blood flow to the kidneys, impairing their ability to filter and excrete waste products such as urea nitrogen. Consequently, BUN levels can rise, making the test a valuable marker for kidney function and a possible indicator of HFrEF severity. Studies have shown that HFrEF patients are at risk for worsened outcomes regarding kidney function [33].

#### 2.2.3. Brain Natriuretic Peptide

BNP is a hormone that the heart’s ventricles produce in response to excessive stretching of heart muscle cells. The BNP test is a critical tool for diagnosing HFrEF, as elevated levels of BNP in the blood indicate heart strain and dysfunction. BNP levels increase when HFrEF develops or worsens, making it a valuable marker for diagnosing and assessing the severity of this condition [34,35]. In the context of HFrEF, BNP testing plays a pivotal role in the initial diagnosis and ongoing management of this condition. Elevated BNP levels have been directly associated with an increased severity of HFrEF and can guide treatment decisions, prognostication, and monitoring of therapy effectiveness. Research demonstrates that patients with HFrEF who show higher BNP levels often face a poorer prognosis, underlining the hormone’s significance as both a diagnostic and a prognostic biomarker [34,35].

### 2.3. Modeling

#### 2.3.1. Passive-Aggressive Learning

The Passive-Aggressive (PA) classifier, inspired by Support Vector Machines, is an online learning algorithm designed for the efficient handling of sequential and streaming data [36]. It processes one data point at a time, updating parameters only when predictions are incorrect or fall within a tolerance margin, hence being *passive* when correct and *aggressive* when incorrect [36]. This mechanism allows it to adapt dynamically to new data while maintaining computational efficiency by not requiring retraining as new data are introduced.

The algorithm’s ability to incrementally update at the individual sample level makes it ideal for time-adaptive and personalized modeling. This dynamic nature was leveraged in this study by fine-tuning the model with patient-specific clinical data across multiple visits, enabling it to better capture personalized trends and improve predictions for each patient. We believe its simplicity and flexibility make it well suited for real-time clinical applications requiring adaptive learning.

We chose to utilize the PA algorithm over other time-series modeling approaches due to its unique suitability for this clinical context. Other time-sensitive methods, including common deep learning approaches (LSTMs, Transformers), Hidden Markov Models (HMMs), etc., often require significantly more training computational resources. In addition, it is not always clear whether these models can adapt to the personalization requirements of incremental learning as accurately as the PA algorithm. In clinical settings, where data arrive sequentially and patient trajectories evolve, utilizing methods that can update as new information becomes available is ideal. The PA algorithm’s ability to update in a streaming manner, rather than requiring full retraining with each new data point, allows a more efficient and responsive representation of each patient’s individual trajectory.

#### 2.3.2. Personalization

**Figure 1:**
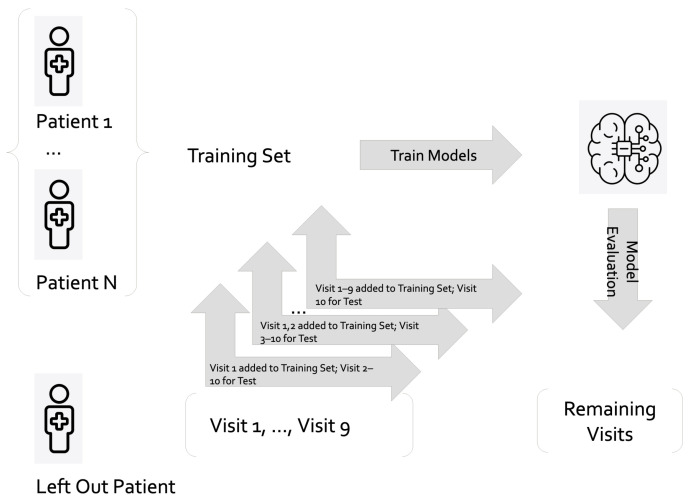
Overview of the LOPOCV training and testing methodology.

In this study, we utilized a PA classifier in a clinical setting, applying the leave-one-patient-out cross-validation (LOPOCV) technique. This approach involves using data from all but one patient to train a generic model, which is then tested on the data from the left-out patient. To personalize the model, we incrementally fine-tuned it using clinical visit data from 1–9 visits of the left-out patient. The remaining data from the left-out patient were subsequently used to evaluate the performance of the personalized model. Figure 1 visually represents the LOPOCV architecture.

### 2.4. Performance Evaluation

To evaluate the performance of the machine learning models in predicting HFrEF severity, we used the accuracy, micro- and macro-averaged recall, precision, F1-score, and one-versus-rest (OVR) AUC scores. Given the multi-class nature of our problem, micro- and macro-averaging provide a more robust evaluation. Micro-averaging aggregates outcomes across all classes, reflecting performance on frequent classes, which makes it effective for datasets with class imbalances. Macro-averaging calculates metrics for each class independently and averages them, highlighting the model’s ability to handle minority classes and ensuring balanced performance across all classes.

## 3. Results

**Figure 2:**
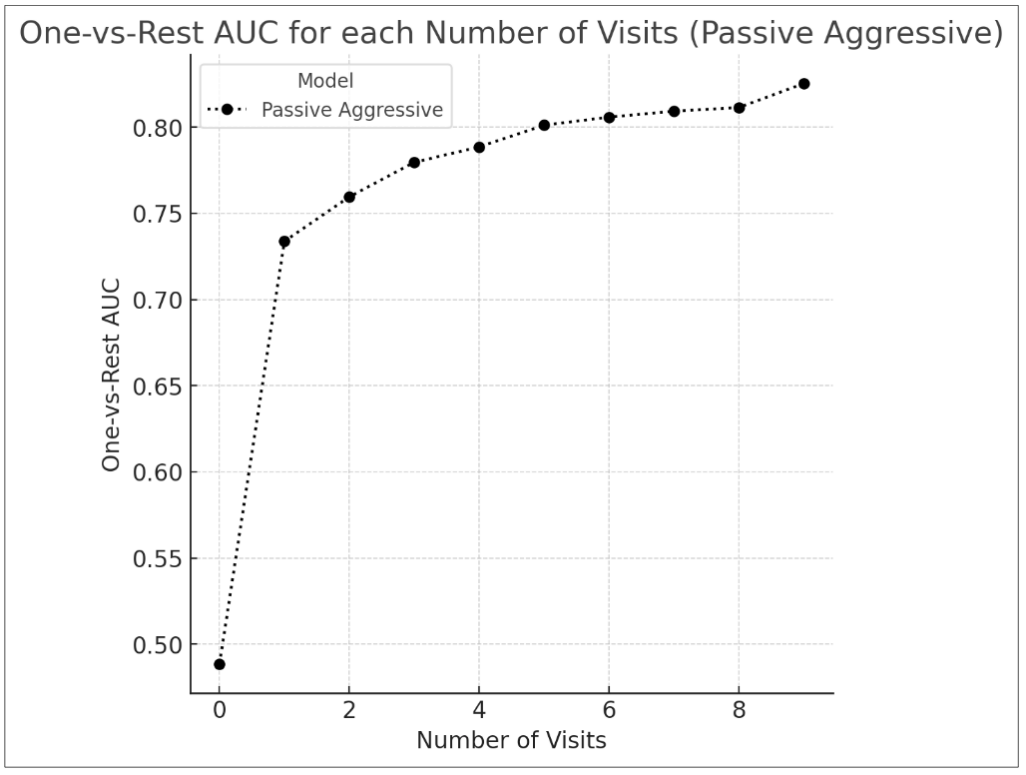
One-vs.-Rest AUC value at each interval for personalization.

Table 3 shows the results over the personalization intervals. Figure 2 provides a visual representation of the OVR-AUC scores for each visit exposed in training. The PA classifier’s performance was evaluated using clinical data from 0–9 visits, with varying levels of personalization. Without personalization (zero visits), the model’s performance was limited, achieving an accuracy of 42.71%, a macro-recall of 33.62%, a micro-recall of 42.71%, a macro-precision of 33.81%, a micro-precision of 42.71%, and an OVR AUC of 0.4884. Introducing personalized data from a single visit significantly enhanced performance, increasing accuracy to 72.98%, macro-recall to 61.42%, micro-recall to 72.98%, macro-precision to 62.27%, micro-precision to 72.98%, and OVR AUC to 0.7338.

The introduction of personalized data led to a noticeable improvement in the model’s performance. When data from a single visit were incorporated, the macro-recall increased from 33.62% to 61.42%, the micro-recall increased from 42.71% to 72.98%, and the OVR AUC increased from 0.4884 to 0.7338. This initial improvement underscores the importance of personalization in capturing individual patient characteristics and disease progression. As more personalized data were introduced, the model’s performance improved. With data from nine visits, the model achieved its peak performance, with a macro-recall of 64.42% and an OVR AUC of 0.8253. This improvement shows the model’s ability to learn and adapt to individual patient health trajectories. Both micro-and macro-metrics followed similar trends, with micro-averaged metrics reflecting the model’s overall predictive strength and macro-averaged metrics highlighting improvements across all classes, including minority ones. The difference in OVR AUC from zero visits to one visit (0.2454) was significantly larger than the difference from one to nine visits (0.0915), highlighting that the initial personalized data had the greatest impact on the model’s performance.

As more personalized data were introduced, performance steadily improved. By nine visits, the model reached its peak, achieving an accuracy of 75.03%, a macro-recall of 64.42%, a macro-precision of 75.14%, and an OVR AUC of 0.8253. Micro- and macro-metrics followed similar trends, with micro-averaged metrics reflecting the model’s overall predictive strength and macro-averaged metrics highlighting improvements across all classes, including minority ones.

The results show the PA classifier’s time-adaptive capabilities. By adapting to individual patient health trajectories, personalized models can capture the dynamic nature of HFrEF and provide more accurate and timely predictions. Incorporating personalized clinical data progressively improved predictive performance, with consistent improvements across micro- and macro-metrics for recall, precision, F1-score, non-averaged accuracy, and AUC. This increase in performance demonstrates the clinical value of incorporating incremental learning algorithms and past patient data. This finding further emphasizes the potential of personalized machine learning models in clinical settings, particularly in managing complex conditions such as HFrEF, where individual variability and disease progression are critical in determining effective treatment strategies over time.

## 4. Discussion

This study underscores the significant practical potential of the Passive-Aggressive (PA) classifier for creating adaptive, patient-specific models in clinical settings, particularly for managing chronic conditions like HFrEF. Our results demonstrate that by sequentially incorporating personalized clinical data, the PA classifier progressively refines its predictive accuracy, as evidenced by consistent improvements across all performance metrics, including accuracy, micro- and macro-averaged precision, recall, F1-score, and OVR AUC. Notably, the most substantial gains were observed when transitioning from a generalized model (no personal data) to incorporating patient-specific information from even a single clinical visit. This highlights the model’s rapid learning capability and its ability to effectively tailor predictions to individual patient trajectories. We believe this modeling advantage offers a new approach to risk stratification in a clinical setting that moves beyond traditional, static risk scores.

The inherent time-adaptive and personalizable nature of the PA classifier makes it exceptionally well suited for the complexities of HFrEF management and other dynamic clinical needs. Unlike static models, it offers a dynamic approach to personalized risk assessment and stratification. As more patient-specific data accrue, the model continuously refines its predictions, enabling proactive care strategies. We believe this will lead to better outcomes for conditions such as HFrEF, where early detection of deterioration and timely adjustments to treatment plans are paramount for improving patient outcomes and reducing hospital readmission. The PA classifier facilitates this by providing increasingly accurate and individualized insights.

For the integration of this model into a clinician’s daily workflow, we envision several practical steps. Personalized and time-adaptive modeling such as this would be excellent in the context of serving as a co-pilot for clinicians. The PA model could be integrated into the Electronic Health Records (EHR) system as a decision-support tool. For example, during a routine check-up, the EHR system could trigger the model to update the patient’s risk prediction upon entering new patient data (e.g., medication changes, laboratory results, vital signs). This updated risk estimation would provide actionable insights and potentially prompt further conversation between clinicians and patients. By continuously analyzing the interplay of various biomedical markers and patient-reported outcomes, the model may be able to identify subtle trends and patterns that might be missed through traditional self-reporting or periodic assessments. This accelerated trend identification could allow clinicians to intervene earlier and more effectively, potentially preventing adverse events.

The methodology employed in this study shows the transformative potential of integrating time-adaptive machine learning into clinical practice. This approach bridges the critical gap between generalized population-based models and the need for precise, individualized predictions, showing a new pathway for more precise medicine. Integrating such adaptive models into clinical workflows and digital health platforms such as remote patient monitoring and wearable technologies holds immense promise. We believe this synergy could further amplify the impact of these models, supporting more efficient, proactive, and personalized management of HFrEF and other chronic diseases. By leveraging the power of incrementally learning algorithms, we can move towards a future where healthcare is more responsive, individualized, and, ultimately, more effective in improving patient lives.

## 5. Conclusions

We introduce a novel framework for time-adaptive HFrEF risk modeling. The model’s accuracy improves with increased historical data, emphasizing its potential for personalized chronic disease management based solely on EHR data. By capturing dynamic changes in patient profiles, it enables timely and tailored interventions. Our findings deepen our understanding of machine learning applications in HFrEF and point toward more adaptive, individualized clinical care. This robust framework holds promise for integration into routine clinical practice, paving the way for personalized data-driven treatment strategies. 

## Figures and Tables

**Table 1:** Biometric measurements from participants.

Demographic Feature	Mean (Standard Deviation)
Age	60.9 (12.3)
BMI	33.8 (9.4)
A1C	6.8 (1.9)
Urea Nitrogen	28.2 (17.7)
BNP	2370.1 (7346.5)

**Table 2:** Table showing the feature set and the category of each feature used for modeling.

Feature	Feature Type
Gender	Categorical
Race	Categorical
Ethnicity	Categorical
Sex at Birth	Categorical
Age at Visit	Continuous
Height	Continuous
Weight	Continuous
Body Mass Index (BMI)	Continuous
Systolic BP	Continuous
Diastolic BP	Continuous
Urea Nitrogen	Continuous
A1C	Continuous
Beta Blocker	Binary
ARB	Binary
CCB	Binary
Diuretic	Binary
Statin	Binary

**Table 3:** Performance metrics for the Passive-Aggressive classifier.

Visits Included	Accuracy	Micro-Recall	Macro-Recall	Micro-Precision	Macro-Precision	Micro-F1	Macro-F1	OVR AUC
0	42.71%	42.71%	33.62%	42.71%	33.81%	42.71%	33.49%	0.4884
1	72.98%	72.98%	61.42%	72.98%	62.27%	73.00%	61.75%	0.7338
2	73.26%	73.26%	62.53%	73.26%	63.09%	73.26%	62.76%	0.7596
3	74.11%	74.11%	62.36%	74.11%	63.11%	74.11%	62.65%	0.7796
4	74.16%	74.16%	61.82%	74.16%	62.39%	74.16%	62.01%	0.7886
5	74.76%	74.76%	63.51%	74.76%	63.96%	74.76%	63.69%	0.8012
6	73.70%	73.70%	61.81%	73.70%	62.51%	73.70%	62.08%	0.8059
7	73.39%	73.39%	62.08%	73.39%	62.40%	73.39%	62.21%	0.8093
8	73.74%	73.74%	61.32%	73.74%	61.81%	73.74%	61.48%	0.8114
9	75.03%	75.03%	64.42%	75.03%	64.57%	75.03%	64.46%	0.8253

## Data Availability

The data used in this study are publicly available to credentialed researchers at https://www.researchallofus.org/ (accessed on 1 March 2024).

## Abbreviations

The following abbreviations are used in this manuscript:

HFrEF	Heart Failure with Reduced Ejection Fraction
PA	Passive-Aggressive
OVR	One-Versus-Rest
LOPOCV	Leave-One-Patient-Out Cross-Validation
BMI	Body Mass Index
BP	Blood Pressure
ARBs	Angiotensin Receptor Blockers
CCBs	Calcium Channel Blockers
BNP	Brain Natriuretic Peptide
